# Wearing masks as a protective measure for children against traffic‐related air pollution: A comparison of perceptions between school children and their caregivers in Ho Chi Minh City, Vietnam

**DOI:** 10.1111/tmi.13923

**Published:** 2023-08-24

**Authors:** Hong H. T. C. Le, Nguyen Truong Vien, Tran Ngoc Dang, Robert S. Ware, Dung Phung, Phong K. Thai, Sarath Ranganathan, Nguyen Nhu Vinh, Phan Hoang Thuy Dung, Huynh Ngoc Thanh, Truong Thi Thuy Dung, To Thi Hien, Peter D. Sly, Pham Le An

**Affiliations:** ^1^ Faculty of Medicine The University of Queensland Brisbane City Queensland Australia; ^2^ Children's Health and Environment Program Centre for Children's Health Research Brisbane City Queensland Australia; ^3^ Department of Environmental and Occupational Health Pham Ngoc Thach University of Medicine Ho Chi Minh City Vietnam; ^4^ Faculty of Public Health University of Medicine and Pharmacy at Ho Chi Minh City Ho Chi Minh City Vietnam; ^5^ Menzies Health Institute Queensland Griffith University Brisbane City Queensland Australia; ^6^ School of Public Health The University of Queensland Brisbane City Queensland Australia; ^7^ Queensland Alliance for Environmental Health Sciences (QAEHS) The University of Queensland Brisbane City Queensland Australia; ^8^ University of Melbourne Melbourne Victoria Australia; ^9^ Centre for the Training of Family Medicine, Faculty of Medicine University of Medicine and Pharmacy at Ho Chi Minh City Ho Chi Minh City Vietnam; ^10^ Grant and Innovation Center University of Medicine and Pharmacy at Ho Chi Minh City Ho Chi Minh City Vietnam; ^11^ University of Science, Vietnam National University Ho Chi Minh City Vietnam

**Keywords:** caregiver, children, health belief, mask, traffic‐related air pollution

## Abstract

**Background:**

Traffic‐related air pollution (TRAP) problems are unlikely to be solved in the short term, making it imperative to educate children on protective measures to mitigate the negative impact on their health. Children and their caregivers may hold differing views on wearing a face mask as a safeguard against air pollution. While many studies have focused on predicting children's health‐protective behaviours against air pollution, few have explored the differences in perceptions between children and their caregivers.

**Objectives:**

To examine this, we conducted a study that compared the health beliefs of two generations and evaluated the factors that influence the use of masks by children to reduce air pollution exposure.

**Methods:**

The study was conducted in 24 secondary schools and involved 8420 children aged 13–14 and their caregivers. We used a Health Belief Model (HBM)‐based instrument containing 17‐item self‐administered health beliefs questionnaires to gather data. The results were analysed using hierarchical logistic regression to determine the probability of children frequently wearing masks to protect against TRAP.

**Results:**

Our study showed both children and caregivers recognised that several factors could influence mask‐wearing among children: discomfort or difficulty breathing while wearing a mask and forgetting to bring a mask when going outside; perceived threats of the poor quality of air and children's respiratory health problems; and cues to mask use (i.e., seeing most of their friends wearing facemasks and ease of finding masks in local stores). However, only children were significantly concerned with public perception of their appearance while wearing a mask. Females were more likely to wear masks, and caregivers with higher levels of education were more likely to encourage their children to wear masks. Children who commuted to schools by walking, biking, or motorbiking were also more accepting of mask‐wearing than those who travelled by car or bus.

**Conclusions:**

Children and their caregivers hold different perceptions of wearing masks to protect against air pollution. Children are more susceptible to social judgements regarding their appearance when wearing a mask.

## INTRODUCTION

Traffic is a significant source of air pollutants in urban areas, hence traffic‐related air pollution (TRAP) has become a significant issue of concern [1]. It arises from vehicle exhausts, secondary pollutants formed in the atmosphere, evaporative emissions from vehicles, and non‐combustion emissions [2]. It contains a mixture of gases and particles, including ground‐level ozone, various forms of carbon, nitrogen oxides, sulphur oxides, volatile organic compounds, polycyclic aromatic hydrocarbons, and fine particulate matter [2]. Exposure to this complex and variable mixture of gases and particles has been linked to a range of adverse health effects across all life stages, from alterations in cellular ecology to mortality [2]. Children, especially those who live near major roads, are particularly vulnerable to the health effects of TRAP [3]. Consequently, measures to minimise children's exposure to TRAP are urgently needed.

While long‐term solutions are being pursued, reducing individual exposure through the use of masks has become a common practice in highly polluted areas. Although the effectiveness of masks in protecting against air pollutants is debated, and masks may not provide sufficient protection in certain situations, they are widely accepted as a personal protective measure against air pollution and can prevent immediate effects of air pollution [4, 5, 6, 7]. Wearing various types of facemasks in public settings has been common practice in Asian countries for decades [8], and public acceptance of such masks is increasing in other countries after the COVID‐19 pandemic [9].

The Health Belief Model (HBM) is a widely used theoretical framework for understanding health behaviours [10]. Our research team developed an HBM‐based instrument to investigate health beliefs related to self‐protective measures by wearing a mask against TRAP exposure [11]. The study questionnaire was based on the following six constructs of the HBM: perceived threats, perceived susceptibility, self‐efficacy, benefits, barriers, and cues to action. Results of the pilot study suggested that individuals who believe in the adverse effects and severity of air pollution (Perceived Threats), feel personally vulnerable to pollution‐related health problems (Perceived Susceptibility), and have confidence in their ability to consistently wear a mask (Self‐efficacy) could be more likely to adopt a protective behaviour such as mask wearing. Perceptions of the benefits and barriers to using masks play a key role in determining the likelihood of successfully maintaining this protective behaviour. In addition, cues to action are necessary to move individuals from accepting masks to actually wearing them. The proposed constructs aimed to predict the level of engagement in wearing masks to mediate the effects of TRAP. However, each of these constructs has a unique contribution towards the adoption of this behaviour which we explore in this study.

**FIGURE 1:**
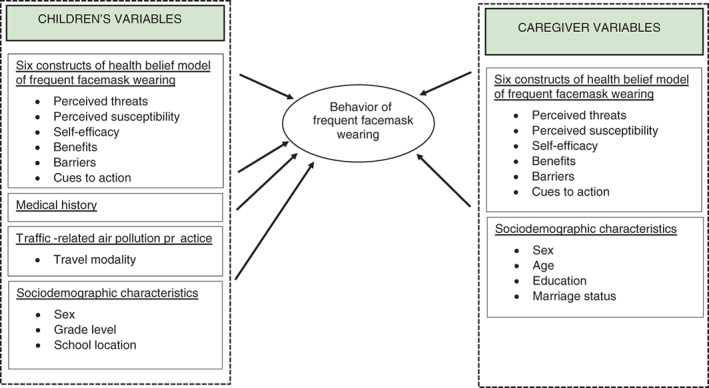
Framework of the study—Conceptual model predicting the behaviour of frequent facemask wearing.

The development of children's behaviours is shaped by the opinions and attitudes of their parents or caregivers. Family factors play a crucial role in forming the health‐protective behaviours of young individuals [12, 13, 14, 15, 16]. Parental support and approval can help children adopt positive attitudes, beliefs, and behaviours that are sustainable. However, in adolescence, children's views may differ from those of their parents, leading to conflicts, which is a normal part of the parent–child relationship [17]. While many studies have focused on identifying components in the model that could be used to predict health‐protective behaviours against air pollution, few studies have explored the differences in perceptions between children and their caregivers [18, 19, 20, 21, 22]. We tested the hypothesis that children and their parents/caregivers may have different perceptions about wearing a facemask to protect against TRAP. The study compared the HBMs of both generations and explored the factors that influence children's adoption of mask use as a preventive measure against TRAP exposure (Figure 1).

## METHODS

### Procedure

**FIGURE 2:**
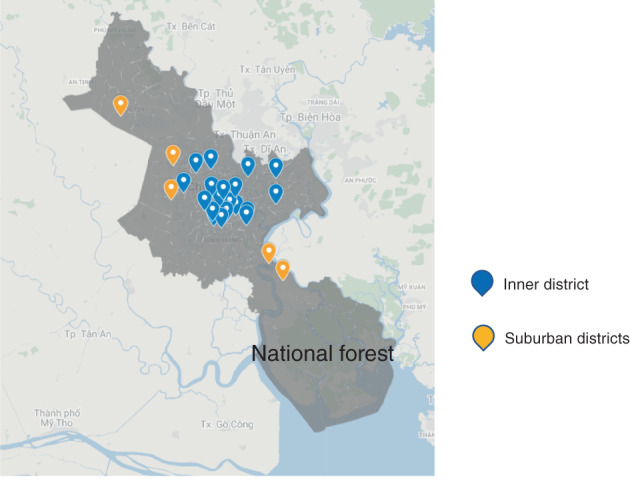
Distribution of selected schools in Ho Chi Minh City [26].

A cross‐sectional survey was conducted in secondary schools in Ho Chi Minh City, Vietnam between November 2019 and February 2020, prior to the outbreak of COVID‐19 in the country. The schools were selected through stratified sampling, with a total of 24 schools chosen from among the 268 public secondary schools located in the 24 districts of Ho Chi Minh City (Figure 2). Seventh and eighth‐grade students (around 13–14 years old) and their caregivers were invited to participate in the survey and complete a questionnaire on their health beliefs regarding mask‐wearing by children to prevent exposure to traffic‐related air pollution. Caregivers received an information sheet and a questionnaire, which they were asked to complete and return to the teachers. The student questionnaire was self‐administered in the classroom, and completed forms were placed in envelopes for privacy. Those who did not wish to participate could return blank forms. The envelopes were only opened by the research team.

### Study location

The study was conducted in Ho Chi Minh City, located in the southeast region of Vietnam. As the economic hub and financial centre of the country, the city is known for its heavy traffic. Results from a 2017 city emission inventory revealed that traffic is the largest contributor to emissions, accounting for approximately 45% or 1813 tons per year [23].

### Study tools

A 17‐item questionnaire with a self‐administered format was used in the study (Supplement A). The content validity, test–retest reliability, and internal consistency of the questionnaire have been previously established and reported [11]. The questionnaire aimed to assess health belief factors related to wearing a facemask to prevent exposure to traffic‐related air pollution. A 3‐point Likert‐type scale, including ‘agree’, ‘disagree’, and ‘unsure’, was used for all scale items. An additional three questions regarding the practice of mask use and three questions regarding sociodemographic characteristics were included to provide more information about participants. Both children and their caregivers were given the same set of health belief questions.

### Ethics

This study was approved by the Board of Ethics in Biomedical Research at the University of Medicine and Pharmacy in Ho Chi Minh City (342/UMP‐BOARD). Permission was obtained from school principals to recruit students and send newsletters to caregivers. Individual student consent was obtained during data collection, after informing them that their participation was completely voluntary. To protect the confidentiality and ensure the right to refuse participation, all questionnaire sheets were placed in an envelope. Children and caregivers were able to leave the answer sheets blank, and they remained sealed until they reached the researchers.

### Data analysis

This study only included data that were valid and obtained from both children and their caregivers. Statistical analysis was performed using Stata version 17.0 (StataCorp, College Station, TX, USA). The two datasets of children and their caregivers were merged if they met all required identification criteria including student ID, class ID, and school ID.

Statistical analyses were conducted in three stages [24]: The first stage consisted of an analysis of internal consistency reliability among items, including the item‐to‐total correlation (a level of 0.30 or higher is acceptable) and the correlation among items. Cronbach's alpha of 0.60 or higher is acceptable (Supplements B and C). Kaiser–Meyer–Olkin (KMO) measures of sampling adequacy and Bartlett's test of sphericity were performed (Supplement D). A KMO value of 0.5 or higher, and a *p*‐value below 0.05 were considered statistically significant. The second stage was an exploratory factor analysis (EFA), to determine the number of factors and percentages of explained variance, which extracted factors according to eigenvalues greater than 1.0, factor loading higher than 0.5, and used processes of principal components factor and orthogonal rotation. The third stage employed confirmatory factor analysis (CFA) to evaluate the validity of the health belief items. A value of factor item loadings at or above the 0.3 level was considered significant in factor analysis [25]. Goodness‐of‐fit indices were used to assess the observed model and the theoretical model (Supplement E).

Hierarchical logistic regression was used to explain the probability of frequently wearing masks among children to protect them from TRAP. The outcome variable was a binary measure of mask usage frequency, with two values indicating ‘often use’ (daily or more than 3 days a week) and ‘not often use’ (less than 3 days a week, or only in some situations or never using a mask). Two models were constructed. In Model 1, only the health belief factors of children and their caregivers were included. In Model 2, socio‐demographic characteristics, TRAP patterns, and medical history were added. These variables were locked in Model 2 in the first step, and then the HBM variables were added in the second step.

## RESULTS

A total of 15,699 questionnaires were distributed to the 24 participating secondary schools. Of these, 15,112 (96.3%) were completed and returned by children, and 8722 (55.6%) by caregivers. Of the 8722 questionnaires returned by caregivers, approximately 8420 (96.5%) contained sufficient valid information (IDs) to be matched with their children's answer sheets. These 8420 sets of matched responses from children and caregivers provided valid data and were included in the analysis.

Table 1 displays the characteristics of the participants. The distribution of male and female participants was equal among children but skewed towards higher numbers of females among caregivers. The ratio of participants from schools in inner areas to those from suburban areas was 80.4%–19.6%. Table 2 presents the medical history of the children. One‐third of the child population reported wheezing or whistling in the past year. Approximately 7% of the children had a history of asthma, while over 45% reported a history of hay fever, and only about 8% reported a history of eczema during their lifetime. Blocked nose, sneezing, and runny nose were the three most frequently reported symptoms reported during the study month. Table 3 presents information on traffic‐related practices, including the means of transportation and mask‐wearing.

**TABLE 1:** Children's and caregivers’ characteristics (*n* = 8420).

Variables	Frequency	Percentage (%)
Children		
Children's sex (*n* = 8406, missing = 14; 0.2%)		
Male	3933	46.8
Female	4473	53.2
Children's grade level		
Grade 7	4475	53.2
Grade 8	3945	46.9
School location (total 24 districts)		
Inner area (19 districts)	6768	80.4
Suburban area (5 districts)	1652	19.6
Caregivers		
Caregivers' sex (missing = 14)		
Male	2410	29.0
Female	5895	71.0
Caregivers' age (*n* = 6586)		
Mean ± SD	42.56 ± 6.54	
Median (IQR)	42 (38–46)	
Caregivers' education level		
Primary school	144	1.7
High school	2477	29.4
College/university graduate	2786	33.1
Postgraduate	2338	27.8
Other	675	8.0
Caregivers' marital status		
Married or living together	7253	86.1
Single	184	2.2
Divorced	536	6.4
Widowed	153	1.8
Other (refused or missing)	294	3.5

**TABLE 2:** Children's medical history (*n* = 8420).

Variables	Frequency	%
Wheezing or whistling in the chest in the last 12 months		
Yes	3101	36.8
No	5319	63.2
Sneezing or runny, or blocked nose when a child did not have a cold or the flu in the last 12 months		
Yes	6765	80.3
No	1655	19.7
Itchy rash in the last 12 months		
Yes	1758	20.88
No	6662	79.1
History of asthma		
Yes	651	7.7
No	7769	92.3
History of hay fever		
Yes	3830	45.5
No	4590	54.5
History of eczema		
Yes	710	8.4
No	7710	91.6
Symptoms in the last month		
Blocked nose	5730	68.1
Sneezing	5538	65.8
Runny nose	5031	59.8
Wet cough	3736	44.4
Itchy nose	3356	39.9
Dry cough	2479	29.4
Itchy eyes	1769	21.0
Wheezing or whistling	1528	18.2
Watery eyes	1505	17.9
Itchy skin	1493	17.7
Itchy rash	877	10.4
Red eyes	676	8.0
Chest pain	567	6.7
Number of symptoms in the last month	4 (2–6) Median (IQR)	
Less than 4 symptoms	3670	43.6
To 6 symptoms	3116	37.0
Over 6 symptoms	1634	19.4

**TABLE 3:** Traffic‐related practices (*n* = 8420).

Variables	Frequency	%
Travel means		
Open‐air vehicle or walking (e.g., motorbike, bicycle, walking)	7030	83.5
Close‐air vehicle (e.g., public bus, car/taxi)	1390	16.5
Trucks often passing by the front of the house (missing = 207, 2.5%)		
Never	1472	17.9
Sometimes	3634	44.2
Often	2009	24.5
Almost every day	1096	13.4
Mask‐wearing during travelling in the street		
Never	1581	18.8
Only on occasions of illness or long trips	1560	18.5
Less than 3 days/week	690	8.2
Three or more days/week	1213	14.4
Daily	3376	40.1

### Exploratory factor analysis and confirmatory factor analysis

Two models of health beliefs were established: the Child Model and the Caregiver Model. The EFA results determined the number of attributes in each model and the percentages of explained variance. The Child Model had four factors consisting of 10 items, which served as indicators of perceived external barriers (3 items in factor 1), perceived threats (3 items in factor 2), self‐barriers (2 items in factor 3), and cues to action (2 items in factor 4). These factors accounted for 58.2% of the observed variance. The Caregiver Model had four factors consisting of 11 items, which served as indicators of perceived external barriers (3 items in factor 1), perceived threats (4 items in factor 2), self‐barriers (2 items in factor 3), and cues to action (2 items in factor 4). These factors accounted for 59.3% of the observed variance (Supplement C).

**FIGURE 3:**
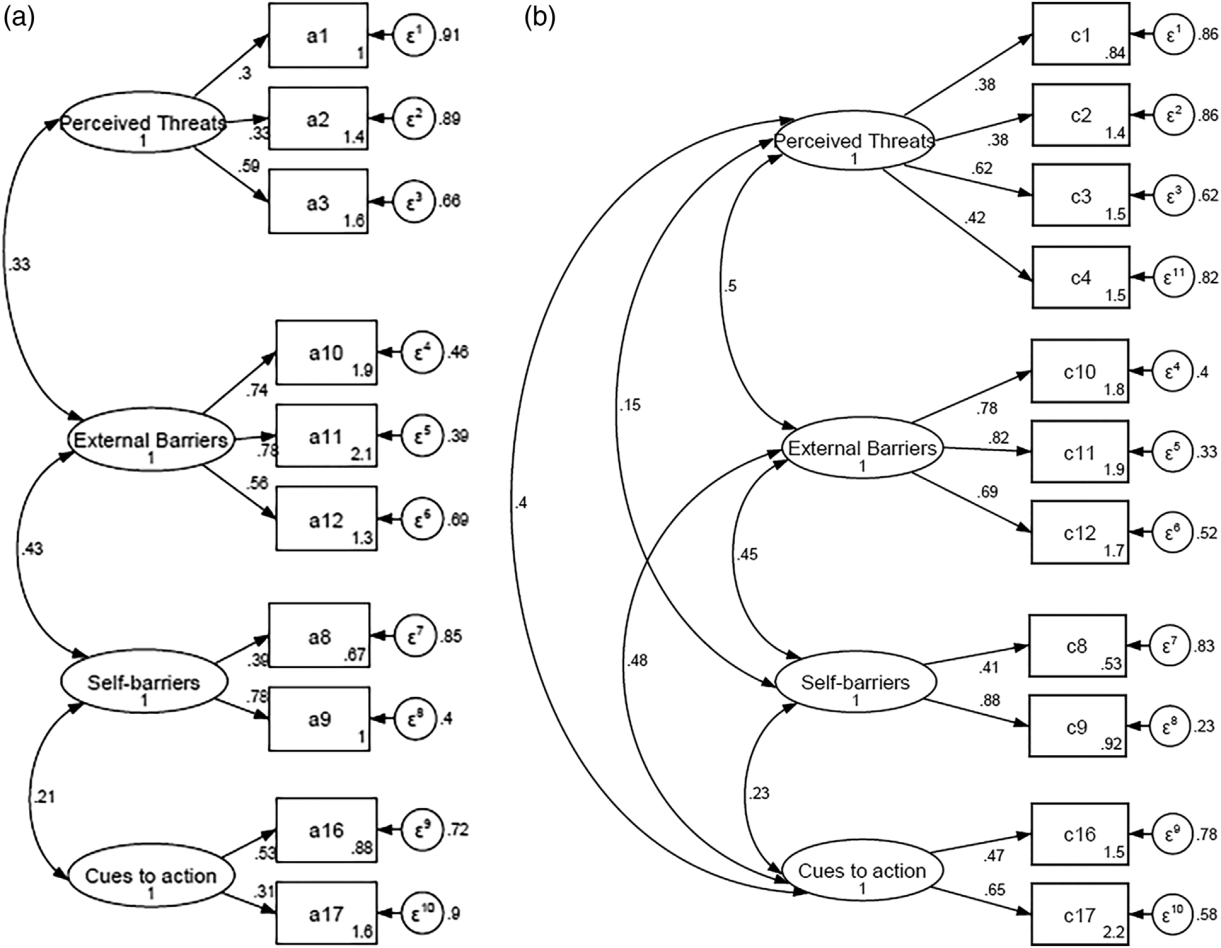
Confirmatory factor analysis for HBM scale (*n* = 8420). Ovals: factors; value in ovals: standardised variances; rectangles: items; values in rectangles: intercepts and random errors of each item; values on one‐way arrows are standardised factor loading; values on two‐way arrows show covariances.

The CFA results confirmed the construct validity of the questionnaire in evaluating the factors of health beliefs in both children and caregivers, presented in Figure 3. The CFA revealed a good correlation of each factor with another in both models with standardised covariances ranging from 0.21 to 0.50. All factor loadings were significant (higher than 0.3 and ranged from 0.30 to 0.88). These results indicated an acceptable to good ability to explain factors relating to the variation of items in the models. Regarding the goodness of fit statistics, both models showed a fair fit to the data, as demonstrated in Supplements E and F.

Hierarchical logistic regression was used to determine the probability of frequent mask use among children as a protective measure against traffic‐related air pollution. First, Model 1 only included the health belief components of both children and their caregivers. As shown in Table 4, the HBM variables were significantly correlated with mask use (*χ* ^2^(8) = 1805.05, *p* < 0.001) and accounted for 16% of the variance in mask utilisation. Next, Model 2 aimed to assess the effect of controlling for socio‐demographic characteristics, traffic‐related practices, and medical history on the predictive value of the HBM components in explaining the mask‐wearing practice. In this model, the socio‐demographic characteristics, traffic‐related practices, and medical history were entered in the first step, followed by the HBM variables in the second step. The results indicated that this model was highly predictive of mask use (*χ* ^2^(8) = 1659.28, *p* < 0.001) and explained 19% of the variance in children's frequent mask use.

**TABLE 4:** Hierarchical logistic regression analysis of Health Belief Model factors and sociodemographic characteristics predicting frequent mask use for air pollution prevention.

Variables	Model 1			Model 2		
	Odds ratio	Odds ratio 95% CI	*p*‐value	Odds ratio	Odds ratio 95% CI	*p*‐value
Children's sex				0.46	0.41–0.52	<0.001
Children's grade level				1.13	1.01–1.27	0.03
School location				0.86	0.74–0.99	0.04
Caregivers' sex				0.88	0.78–1.01	0.07
Caregivers' age				1.00	0.99–1.01	0.35
Caregivers' marital status				0.92	0.86–1.00	0.04
Caregivers' education level				1.13	1.07–1.21	<0.001
Method of open‐air travel (motorbike, bicycle, or walking)				1.51	1.23–1.77	<0.001
Frequency of trucks passing by the house				1.03	0.97–1.09	0.40
Wheezing or whistling in the last 12 months				1.03	0.90–1.17	0.70
Sneezing or runny, or blocked nose when not due to cold or flu in the last 12 months				1.11	0.94–1.30	0.22
Itchy rash in the last 12 months				1.12	0.96–1.29	0.14
History of asthma				1.17	0.94–1.45	0.15
History of hay fever				1.09	0.97–1.24	0.16
History of eczema				0.92	0.75–1.13	0.44
Number of symptoms in the last month				1.02	0.99–1.04	0.25
Health Belief Model constructs						
Children's external barriers	1.30	1.12–1.51	<0.001	1.21	1.01–1.44	0.04
Children's perceived threats	1.54	1.31–1.81	<0.001	1.56	1.28–1.88	<0.001
Children's self‐barriers	6.53	5.66–7.52	<0.001	6.64	5.62–7.85	<0.001
Children's cues to action	1.20	1.04–1.37	0.01	1.09	0.92–1.28	0.33
Caregivers' external barriers	0.90	0.78–1.05	0.18	0.83	0.69–0.99	0.04
Caregivers' perceived threats	1.25	1.05–1.49	0.01	1.01	0.81–1.26	0.93
Caregivers' self‐barriers	3.26	2.81–3.77	<0.001	3.51	2.95–4.18	<0.001
Caregivers' cues to action	1.25	1.08–1.46	0.003	1.17	0.97–1.40	0.10
	Model *χ* ^2^(8) = 1805.05, *p* < 0.001, Pseudo *R* ^2^ = 16%			Model *χ* ^2^(8) = 1659.28, *p* < 0.001, Pseudo *R* ^2^ = 19%		

Abbreviations: 95% CI, 95% confidence interval; *χ* ^2^, chi‐squared test.

Regarding the HBM constructs, the results showed that both children and caregivers who perceived the health threats posed by air pollution were more likely to use masks to protect children from TRAP. However, children were less likely to wear masks if their caregivers perceived barriers to their children wearing masks. The findings also revealed that children who perceived external barrier factors, such as negative perceptions from peers or friends about wearing a mask, were significantly less likely to wear facemasks. However, this external barrier factor did not have an impact on the perception of caregivers.

## DISCUSSION

The current study produced two models that examine children's and their caregivers' health beliefs in wearing masks to prevent exposure to TRAP. This study is unique in that it combines the health perceptions and characteristics of both children and their caregivers to better understand the factors that influence the practice of mask‐wearing. Data was collected from 8420 pairs of children and their caregivers prior to the start of the first COVID‐19 pandemic. The final HBM on the likelihood of wearing masks to prevent TRAP exposure identified four key segments: perceived external barriers, self‐barriers, perceived threats, and cues to action. The analysis of the interaction between health belief factors and mask use indicated that the most important factor limiting children's adoption of protective health behaviour was their own beliefs about barriers to wearing masks. Our results suggest that both children and their caregivers perceive similar self‐barriers, perceived threats, and cues to action. However, only children are concerned about external barriers, particularly regarding how the public perceives them if they adopt the recommended health behaviour.

Both teenagers and their caregivers in the study perceived that less frequent wearing of a mask to protect against TRAP among children was primarily due to discomfort or difficulty breathing while wearing a mask or forgetting to take a mask when going outside. These findings are in line with previous studies, which have shown that barriers are among the strongest predictors of preventive health actions across different behaviours [27, 28, 29]. For example, a study among the Korean population found that physical barriers to wearing a mask, rather than cost‐related barriers, diminished the likelihood of mask‐wearing [29]. On the other hand, a study of Chinese adults living in smog‐affected areas found that perceived barriers were the weakest predictor of mask‐wearing behaviours while perceived threats were superior factors to their HBM [30]. The discrepancy between the Chinese study and ours, which has similar findings to the Korean study, supports the hypothesis that when a threat is invisible, people are less likely to act, and in the context of invisible air pollution or climate change, people are more likely to be influenced by barriers to taking protective action, such as covering their faces. Nevertheless, in the case of extreme and visible risk events, such as smog or wildfire, their internal barriers may be less important and perceptions of threat may drive their protective behaviours [12, 30, 31].

Another important finding from the current study was that perceived threats and cues to action played a significant role in determining frequent mask‐wearing. Participants, including both children and their caregivers who perceived threats of the poor quality of air and children's respiratory health problems, were more likely to wear a face mask. As previously mentioned, for individuals to recognise the dangers of air pollution, they need to be aware of its presence through direct observation or sensory perception. For example, in the study by Claeson et al. among residents living near a biofuel facility that emitted odorous substances, air pollution risks were well perceived [32]. If air pollutants can be detected by the senses (such as smell or sight) without the use of any measuring devices, then the air quality is considered to be very poor [33]. However, by the time air pollution becomes visible, adverse health effects are likely to have already occurred, and wearing a mask at that point may be too late. In our final HBM, two cues to action were found to be significant: seeing most of their friends wearing facemasks outdoors and the ease of finding masks in local stores. Children are more likely to take a recommended action if it is widely accepted by their peers [34, 35].

A notable difference in health belief constructs between children and their caregivers in the present study was external barriers. Many studies provide evidence of the significant influence of children's peers and social networks [34, 35]. The current findings also align with earlier observations [34, 35, 36, 37] but they offer more specific insight into preventive health behaviour, particularly the use of face masks in response to TRAP. While mature individuals are less sensitive to social judgements, the perception of peers considerably influences behaviour choices during adolescence. In the current study, the young population who reported a higher level of perceived external obstacles showed a lower level of commitment to pollution‐protective behaviours, particularly wearing masks. Although the study was conducted in an Asian context where wearing face shields is very common in public settings, the children respondents were still concerned about appearing weird, sick, or weak while covering their faces. Another possible explanation for this finding could be related to the adolescent perception of invincibility. It is typical for an adolescent to have a sense of being invincible or immune to health risks [38]. This mindset could prevent teens from seeking or taking protective behaviours if they feel it makes them less powerful. Therefore, these findings should be taken into account when developing health promotion interventions for this age group.

Notably, this study investigated the relationship between children's and caregivers' sociodemographic characteristics and children's medical records, and the frequency of mask use to prevent TRAP. Female teenagers were more likely to wear masks than males, which is consistent with the findings of previous studies [29]. Caregivers with a higher level of education were also more likely to support mask‐wearing by their children, which further supports the idea that education is positively associated with healthy behaviours. The daily means of transportation from home to school was found to be a critical factor influencing the intention to wear a mask among the study respondents. Children who travelled by open‐air vehicles such as motorbikes and bicycles or who walked to school were more accepting of putting a mask on. Wearing masks in public has been common in Asian countries for a long time [8], as these modes of transportation expose individuals to more TRAP. Unfortunately, the current study revealed that health history was not associated with the practice of wearing a mask. In a pilot study with a smaller sample size a relationship was found between personal respiratory symptoms and mask use. However, with a larger sample size and wider modelling, this association was not confirmed. Therefore, this relationship is still a matter of debate and requires further investigation.

This study primarily focused on the main reason for wearing masks, which is to protect against traffic‐related air pollution. However, our published qualitative pilot study explored additional benefits of wearing masks among children, taking into account not only air pollution but also other factors [11]. The additional benefits of covering the face with a mask could include protecting facial skin, avoiding unpleasant smells or sunlight, and complying with parental requests. Gaining a better understanding of these additional reasons will contribute to a comprehensive understanding of children's behaviours and facilitate the development of mask‐wearing campaigns for children.

### Limitations and strengths

This study has several limitations that need to be acknowledged. First, the exclusion of 6692 unmatched data points of children from the analysis can introduce a selection bias into this study. Nevertheless, we found the percentage of children frequently wearing facemasks did not significantly differ between those with caregivers included in this study (54.5%) and those excluded from this study (49.7%). Second, the responses from caregivers were based on volunteer participation, so the adults who participated in the study may have a higher interest in environmental matters, potentially leading to an overestimation of their perceptions and behaviours towards air pollution. Lastly, the current study reflected the perceptions and behaviours prior to the COVID‐19 pandemic, which has widely promoted mask‐wearing globally. As a result, the combined effects of air pollution and COVID‐19 on the practice of wearing masks have not been evaluated.

Despite these limitations, this study is a preliminary effort to integrate the health beliefs and characteristics of both children and their caregivers into a model to examine the actual facilitators of mask‐wearing. The research provides additional insights into the role of caregivers in promoting pollution‐avoiding behaviours among children. Furthermore, the study was based on a relatively large sample size with a high response rate, yielding more accurate estimates of population values.

### Implication

The findings of this study have practical implications for public health researchers and practitioners working in the context of air pollution. First, to encourage mask use, efforts should be focused on helping subjects overcome perceived barriers. Many masks are currently being produced with special features for comfort and convenience. This information should be disseminated to motivate individuals to engage in recommended protective behaviours. Second, peer influence plays a crucial role in shaping the healthy behaviours of young people. Child‐targeted intervention programs should involve peer participation or peer educators to increase the effectiveness of these programs. Children are often concerned about public perceptions of their appearance, hence making mask‐wearing normal or fashionable could help to increase mask acceptance. Lastly, air pollution is often invisible and has long‐term rather than immediate consequences; accordingly, education will remain key to change perceptions and then behaviours.

## CONCLUSION

Health perceptions regarding wearing a facemask to prevent exposure to TRAP differ between children and their caregivers. Children perceived self‐barriers as the major hindrance to their adoption of health‐protective behaviour in response to air pollution, particularly wearing a face mask. Children were more influenced by social judgements of their appearance when covering their faces than their caregivers: peer influence is important in children's mask usage. These findings have significant implications for health education and intervention programs targeting children.

## FUNDING INFORMATION

This work was supported by a collaborative research project between Vietnam National Foundation for Science and Technology Development (NAFOSTED—Grant ID: NHMRC.108.03‐2018.04) and National Health and Medical Research Council (NHMRC—Grant ID: APP1155241).

## Supporting information


**Data S1.** Supporting Information.
