# Supplementary material for: Wearing masks as a protective measure for children against traffic‐related air pollution: A comparison of perceptions between school children and their caregivers in Ho Chi Minh City, Vietnam
Source: Trop Med Int Health. 2023 Aug 24;28(9):753–62. doi: 10.1111/tmi.13923 (PMC10946546; doi:10.1111/tmi.13923)
Supplement: Supplementary file 1 — Data S1. Supporting Information. [file TMI-28-753-s001.pdf]

Supplement A: Full questionnaires for children and caregivers

ID in Children model	ID in Caregiver model	Questions for children	Questions for caregiver
a1	c1	During the last month, the air quality in my area is acceptable.	During the last month, the air quality in my child’s area is acceptable.
a2	c2	Breathing polluted air while I am on the street can lead to death or dangerous diseases such as cancer.	Breathing polluted air while my child is on the street can lead to death or dangerous diseases such as cancer.
a3	c3	My lung is healthy, so I won’t be affected by air pollution.	My child’s lung is healthy, so he/she won’t be affected by air pollution.
a4	c4	I easily get sneezing, runny, or blocked nose when I breathe polluted air from traffic	My child easily gets sneezing, runny, or blocked nose when he/she breathes polluted air from traffic
a5	c5	Wearing a paper facemask/normal medical mask cannot protect me against air pollution while I am on the street.	Wearing a paper facemask/normal medical mask cannot protect my child against air pollution while he/she is on the street.
a6	c6	Wearing respirators such as N95 can protect me against air pollution while I am on the street.	Wearing respirators such as N95 can protect my child against air pollution while he/she is on the street.
a7	c7	Washing a facemask frequently is a waste of my time	Washing a facemask frequently is a waste of my child’s time
a8	c8	I often forget to bring a facemask with me when going out	My child often forgets to bring a facemask with her/him when going out
a9	c9	I feel discomfort or difficulty breathing if I am wearing a facemask.	My child feels discomfort or difficulty breathing if he/she is wearing a facemask.
a10	c10	People will think I am weird if I am wearing a facemask.	People will think my child is weird if he/she is wearing a facemask.
a11	c11	I look weaker if I am wearing a facemask.	My child looks weaker if he/she is wearing a facemask.
a12	c12	People think I am sick if I am wearing a facemask.	People think my child is sick if he/she is wearing a facemask.
a13	c13	I can always wear a facemask even if I don’t know whether it can protect me.	My child can always wear a facemask even if he/she doesn’t know whether it can protect her/him.
a14	c14	I can always wear a facemask for a long while even if I feel uncomfortable	My child can always wear a facemask for a long while even if he/she feels uncomfortable
a15	c15	If I am wearing a mask, it is because my parent(s)/ older family members ask me to do this.	If my child is wearing a mask, it is because I or our older family members ask her/him to do this.
a16	c16	Most of my friends are wearing facemasks while they are on the street.	Most of my child’s friends are wearing facemasks while they are on the street.

a17	c17	It is easy to find a facemask store in my area	It is easy to find a facemask store in my child’s area

Supplement B: Cronbach's alpha analysis of the HBM scale (n=8420)

ID in Children model	ID in Caregiver model	Items*, **	Children' beliefs				Caregivers' beliefs			
			Original version		Final version		Original version		Final version	
			Item-test correlation	alpha (if item excluded)	Item-test correlation	alpha (if item excluded)	Item-test correlation	alpha (if item excluded)	Item-test correlation	alpha (if item excluded)
a1	c1	The air quality in my area is acceptable.	0.315	0.622	0.321	0.631	0.343	0.689	0.353	0.701
a2	c2	Breathing polluted air can be dangerous	0.336	0.617	0.345	0.625	0.365	0.686	0.378	0.697
a3	c3	Not be affected by air pollution.	0.392	0.607	0.408	0.613	0.470	0.672	0.483	0.681
a4	c4	Easily get respiratory symptoms	0.318	0.622	0.325	0.630	0.409	0.680	0.413	0.691
a5	c5	Wearing a facemask cannot protect against air pollution	0.330	0.619	0.340	0.627	0.257	0.695	**excluded**	
a6	c6	Wearing respirators (N95) can protect against air pollution	0.353	0.615	0.361	0.622	0.33	0.691	0.330	0.704
a7	c7	Washing a facemask wastes time	0.428	0.603	0.432	0.611	0.372	0.685	0.389	0.695
a8	c8	Often forget to bring a facemask	0.403	0.606	0.391	0.616	0.311	0.689	0.349	0.696
a9	c9	Feel discomfort or difficulty breathing while wearing masks	0.490	0.592	0.504	0.597	0.455	0.675	0.505	0.679
a10	c10	Think weird while wearing masks	0.519	0.588	0.542	0.590	0.625	0.65	0.650	0.656
a11	c11	Look weaker while wearing masks	0.544	0.585	0.565	0.588	0.629	0.652	0.653	0.656
a12	c12	Think sick while wearing masks	0.479	0.594	0.493	0.599	0.576	0.658	0.600	0.663
a13	c13	Always wear a facemask even not sure of the effectiveness	0.336	0.618	0.322	0.629	0.444	0.676	0.436	0.690
a14	c14	Always wear a facemask even feel uncomfortable	0.248	0.627	**excluded**		0.303	0.691	**excluded**	
a15	c15	Parents/elders ask to wear masks	0.251	0.629	**excluded**		0.282	0.696	**excluded**	
a16	c16	Friends are wearing facemasks	0.375	0.612	0.382	0.620	0.427	0.677	0.421	0.690
a17	c17	Easy to find a facemask	0.355	0.613	0.373	0.618	0.443	0.674	0.436	0.685
		**Overall alpha**		**0.625**		**0.631**		**0.692**		**0.701**

*All items have been recoded to posit the hypothesis that the items positively predict the behavior of mask-wearing
** Full questions were shown in Supplement A

Supplement C: Principal-component factors analysis and rotated factor loadings (n=8420)

ID in Children model	ID in Caregiver model	Items	Children' beliefs #				Caregivers' beliefs #			
			Factor 1	Factor 2	Factor 3	Factor 4	Factor 1	Factor 2	Factor 3	Factor 4
a1	c1	The air quality in my area is acceptable.		0.6731				0.6637		
a2	c2	Breathing polluted air can be dangerous		0.6606				0.6109		
a3	c3	Not be affected by air pollution.		0.6405				0.622		
	c4	Easily get respiratory symptoms						0.5586		
a8	c8	Often forget to bring a facemask			0.8554				0.8772	
a9	c9	Feel discomfort or difficulty breathing while wearing masks			0.7012				0.7145	
a10	c10	Think weird while wearing masks	0.8223				0.8220			
a11	c11	Look weaker while wearing masks	0.8207				0.8456			
a12	c12	Think sick while wearing masks	0.7149				0.8049			
a16	c16	Friends are wearing facemasks				0.7910				0.8224
a17	c17	Easy to find a facemask				0.7102				0.7368
**Eigenvalue**			**2.45013**	**1.25047**	**1.08203**	**1.04143**	**2.33479**	**1.54409**	**1.32405**	**1.31714**
**Explain proportion**			**24.5%**	**12.5%**	**10.8%**	**10.4%**	**21.2%**	**14.0%**	**12.0%**	**12.0%**
**Cumulative explain proportion**			**58.2%**				**59.3%**			

Factor loadings less than 0.5 were hidden

Supplement D: Test of assumption for Factor analysis (n=8420)

Factor test	Childrens' belief Results	Caregivers' belief Results
**Kaiser-Meyer-Olkin Measure of Sampling Adequacy**	0.7640	0.804
**Bartlett test of sphericity**		
Chi-square	11887.868	17433.997
Degrees of freedom	105	91
p-value	<0.001	<0.001

Supplement E: Goodness of fit statistics (n=8420)

Fit statistic	Values		Expected values
	Children model	Caregiver model	
**Likelihood ratio test for model vs. saturated comparison**			
Chi-square	160.803	244.937	
p-value	<0.001	<0.001	>0.05
**Likelihood ratio test for baseline vs. saturated comparison**			
Chi-square	9414.393	15422.885	
p-value	<0.001	<0.001	>0.05
**Population error**			
Root mean square error of approximation (RMSEA)	0.020	0.025	<0.05
RMSEA 90% CI, lower bound	0.017	0.022	
RMSEA 90% CI, upper bound	0.023	0.029	<0.08
p-close *(H_0: Probability RMSEA ≤ 0.05)*	1.000	1.000	>0.05
**Baseline comparison**			
Comparative fit index (CFI)	0.987	0.987	>0.9
Tucker-Lewis index (TLI)	0.981	0.981	Close to 1
**Size of residuals**			
Standardized root mean squared residual (SRMR)	0.016	0.019	<0.08
Coefficient of determination (CD)	0.954	0.987	Close to 1

Supplement F: Factors description

Factors	Name of factors	Children model		Caregiver model	
		Mean ± SD	95% CI	Mean ± SD	95% CI
Factor 1	External barriers	0.74 ± 0.35	0.74 – 0.75	0.76 ± 0.36	0.75 – 0.77
Factor 2	Perceived Threats	0.63 ± 0.32	0.63 – 0.64	0.61 ± 0.30	0.60 – 0.62
Factor 3	Self-Barriers	0.41 ± 0.39	0.40 – 0.42	0.34 ± 0.38	0.33 – 0.35
Factor 4	Cues to action	0.58 ± 0.36	0.57 – 0.59	0.76 ± 0.34	0.75 – 0.77
